# The most common RNF43 mutant G659Vfs*41 is fully functional in inhibiting Wnt signaling and unlikely to play a role in tumorigenesis

**DOI:** 10.1038/s41598-019-54931-3

**Published:** 2019-12-06

**Authors:** Jianghua Tu, Soohyun Park, Wangsheng Yu, Sheng Zhang, Ling Wu, Kendra Carmon, Qingyun J. Liu

**Affiliations:** Texas Therapeutics Institute and The Brown Foundation Institute of Molecular Medicine, The University of Texas Health Science Center at Houston, 1825 Pressler St., Houston, Texas USA

**Keywords:** Cancer genomics, Oncogenes

## Abstract

RNF43 is an E3 ligase that inhibits Wnt signaling by ubiquitinating Wnt receptors for degradation. It is mutated in various cancer types with the most recurrent mutation being the frameshift G659Vfs*41 with frequencies of ~5–8% in colon, stomach and endometrial cancers. This mutation, a deletion of G in a 7-G repeat, has been assumed to encode an inactive enzyme that would lead to increased Wnt signaling and drive tumorigenesis, yet no functional characterization has been reported. We analyzed the distribution of G659Vfs*41 and its association with other cancer gene mutations, and found that the mutation occurred nearly exclusively in tumors with low expression of the DNA mismatch repair gene MLH1. Mutant RNF43-G659Vfs*41 was no different from wild type RNF43 in expression, stability, localization, R-spondin binding, and inhibition of Wnt signaling. No dominant negative activity of the mutant was observed. Colon tumors with RNF43-G659Vfs*41 had low Wnt/β-catenin signaling and were frequently mutated in BRAF. A colon cancer cell line with RNF43-G659Vfs*41 and BRAF-V600E mutations was sensitive to activation of Wnt/β-catenin signaling. These findings suggest that the frequent occurrence of RNF43-G659Vfs*41 may result from error-prone replication of the 7-G repeat in MLH1-deficient tumors and that the mutation itself does not inactivate enzyme.

## Introduction

RNF43, a member of the RING finger E3 ubiquitin ligase family, was originally identified as an oncoprotein with upregulated expression in colon cancer^1^. It is a type I integral membrane protein with an extracellular domain of 175 amino acid (AA) residues, a single transmembrane domain, and an intracellular region of 565-AA residues with the RING finger E3 ligase domain (AA270-316) immediately following the transmembrane domain. A series of subsequent studies revealed that RNF43 and its related paralog ZNRF3 ubiquitinate the frizzled (FZD) family of Wnt receptors for degradation and thus serve as negative regulator of Wnt signaling^2–5^. Meanwhile, genome-wide sequencing of tumor samples uncovered that RNF43 and ZNRF3 were mutated in a wide variety of tumor types, with relatively high frequency (near 10%) found in pancreatic^6^, uterine endometrial^7^, stomach^8^, and colon cancers^7,9–14^. In the COSMIC cancer database, approximately half of the 1,036 mutations of RNF43 identified from all cancer types are either non-sense or frameshift mutations^15^. Some of the recurrent non-sense mutations of RNF43, such as R132x and R145x, are expected to produce non-functional proteins due to the lack of the E3 ligase domain. Also, some missense mutations in the RING domain were shown to have lost activity in Wnt signaling^4,16^. In pancreatic cancer, RNF43 mutations located within or upstream the RING domain were identified and cell lines with such mutations were shown to be sensitive to inhibitors of Wnt ligand secretion^17^. In colon cancer, RNF43 mutations were largely exclusive with APC mutation^7^. All these observations led to a seemingly obvious conclusion that nonsense and frameshift RNF43 mutations are loss-of-function mutations that would lead to tumorigenesis in a significant subset of gastrointestinal and endometrial cancers due to increased Wnt signaling^7,14^. Inhibition of Wnt ligand production is purported to provide an effective treatment for tumors with RNF43 non-sense or frameshift mutations^7,14^.

Remarkably, the single most recurrent mutation of RNF43 is deletion of a G-C base-pair (bp) in a seven G repeat near the 3′ end of its open reading frame (nucleotide 1969–1976), accounting for approximately half of all RNF43 mutations detected in colon, stomach, and endometrial cancer with an overall frequency of 5–8%^7,14,15^. The frameshift, designated G659Vfs*41, truncates the enzyme at Gly659 and shifts the reading frame to add a neopolypeptide of 41-AA^15^. The mutation removes the C-terminal 123-AA region that is far downstream of the E3 ubiquitin ligase domain^4,5^. If and how RNF43-G659Vfs*41 will affect its enzyme activity in regulation of Wnt signaling has never been reported. Nevertheless, the mutation has always been proposed to be loss-of-function and play a role in colon cancer formation/progression due to disinhibition of Wnt signaling^7,14^. Both RNF43 and ZNRF3 ubiquinate lysine residues of FZD receptors for endocytosis and degradation^2–5^. The process is suggested to occur following the activation of Wnt signaling in which the E3 ligases interact with the disheveled (DVL) protein bound to the Wnt signaling complex to catalyze ubiquitination of FZD^4,5^. However, ubiquitination and degradation of FZD were shown to happen without concomitant activation of Wnt signaling, at least with recombinant enzymes and receptors^4^. E3 ubiquitin ligase activity of RNF43 and ZNRF3 can be inhibited by R-spondins, a group of four related secreted proteins (RSPO1–4) that strongly potentiate Wnt signaling and drive tumorigenesis when over-expressed by gene fusion or other mechanisms^18,19^. Here we show that the RNF43-G659Vfs*41 mutant is actually fully functional in promoting FZD degradation and inhibiting Wnt signaling. In the three cancer types with highest incidences of G659Vfs*41, nearly all tumors with this mutation had low expression of MLH1, a major player of DNA mismatch repair. Furthermore, in colon cancer, RNF43-G659Vfs*41 mutation is most frequently associated with BRAF-V600E mutation and low Wnt/β-catenin signaling. Intriguingly, we found that a colon cancer cell line with RNF43-G659Vfs*41 and BRAF-V600E mutations was sensitive to activation of Wnt signaling *in vitro*. Overall, these findings suggest that RNF43-G659Vfs*41 does not inactivate the enzyme and its relatively common occurrence in MLH1-deficient tumors is most likely a result of error-prone replication of a 7-G repeat.

## Results

### RNF43-G659Vfs*41 is found nearly exclusively in cancers with low MLH1 expression

**Figure 1:**
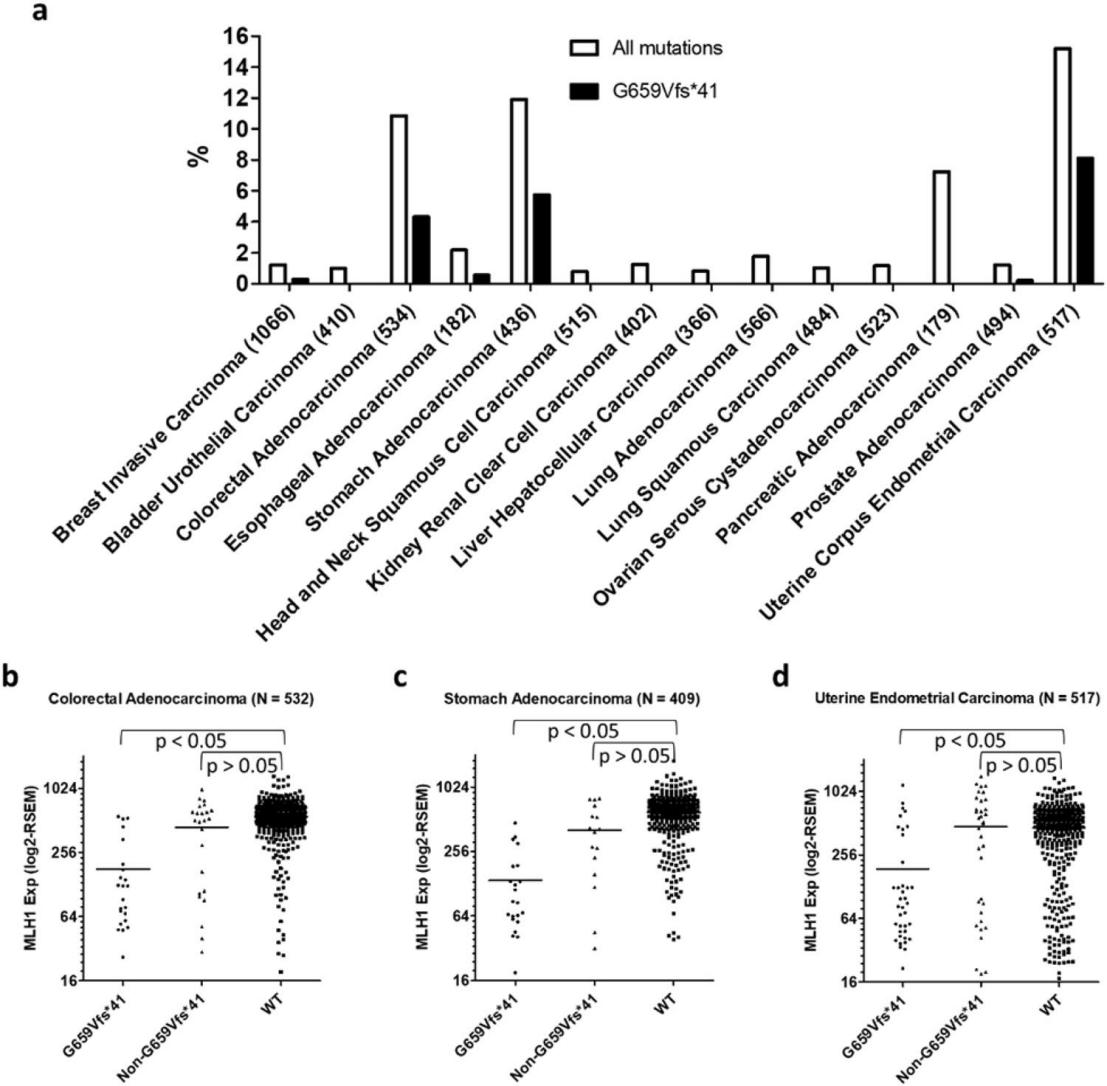
RNF43-G659Vfs*41 is most common in colon, stomach, and endometrial cancers and the mutation is strongly associated with low MLH1 expression. (**a**) Distribution of RNF43 mutations in all major types of solid tumors. (**b**–**d**) Scatter plot of MLH1 expression in relation to mutation status of RNF43 in colorectal cancer (**b**), stomach cancer (**c**) and endometrial cancer. (**d**) p values were calculated using one-way ANOVA followed by Dunn’s multiple comparison test.

We first systematically analyzed RNF43 mutations identified in all major types of solid tumors in the TCGA database using cBioPortal^20,21^. Colon, endometrial, pancreatic, and stomach cancers showed the highest incidences of all RNF43 mutations with frequencies of 10.9%, 15.2%, 7.3%, and 11.9%, respectively (Fig. 1a). In colon, endometrial, and stomach cancers, G659Vfs*41 accounted for 40% (23/81), 53% (42/79), and 48% (25/77), respectively, of all RNF43 mutations, whereas not a single G659Vfs*41 mutation (0/13) was found in pancreatic cancer (p < 0.05 vs any of the other three cancer types, Fisher’s exact test). The only other cancer type with G659Vfs*41 mutation being identified more than once is breast cancer (3/16 RNF43 mutations in 1066 cases). These data indicate that RNF43-G659Vfs*41 occurred nearly exclusively in colon cancers, endometrial, and stomach cancers.

Previously, it was reported that RNF43 mutations were frequently associated with high microsatellite instability (MSI-H) in colon and stomach cancer^7,11,14^. We examined the relationship between RNF43 mutations and MLH1 expression in this TCGA cohort as low expression of MLH1 due to methylation is the most common cause of MSI-H^22^. In colon cancer, RNF43-G659Vfs*41 tumors showed a much lower expression of MLH1 when compared to those of RNF43-WT tumor (mean RNA-seq RSEM value of 179 vs 553, p < 0.05) (Fig. 1b). In contrast, tumors with non-G659Vfs*41 mutations in RNF43 showed no significant difference with those of WT in MLH1 expression (mean RNA-seq RSEM value of 440 vs 553, p > 0.05) (Fig. 1b). Similar data were found in endometrial and stomach cancers (Fig. 1c,d, Supplementary Fig. S1a–c). In pancreatic cancer in which RNF43 was mutated at relative high frequency except that no G659Vfs*41 mutation was identified, none of the tumors had low MLH1 expression (Supplementary Fig. S1d). Furthermore, of all solid tumors, only colon, stomach, and uterine endometrial cancers have a significant subset of tumors with low MLH1 expression (http://firebrowse.org/viewGene.html?gene = mlh1). Taken together, these results unequivocally show that RNF43-G659Vfs*41 mutation in colon, endometrial and stomach cancers primarily occurred in tumors with low MLH1 expression and presumably defective DNA mismatch repair, suggesting that the seven G repeat (bp1969-1976) of RNF43 coding sequence is highly prone to single bp deletion in the absence of proficient DNA repair.

### No discernable difference between RNF43-G659Vfs*41 and wild type in cellular localization and RSPO-binding

**Figure 2:**
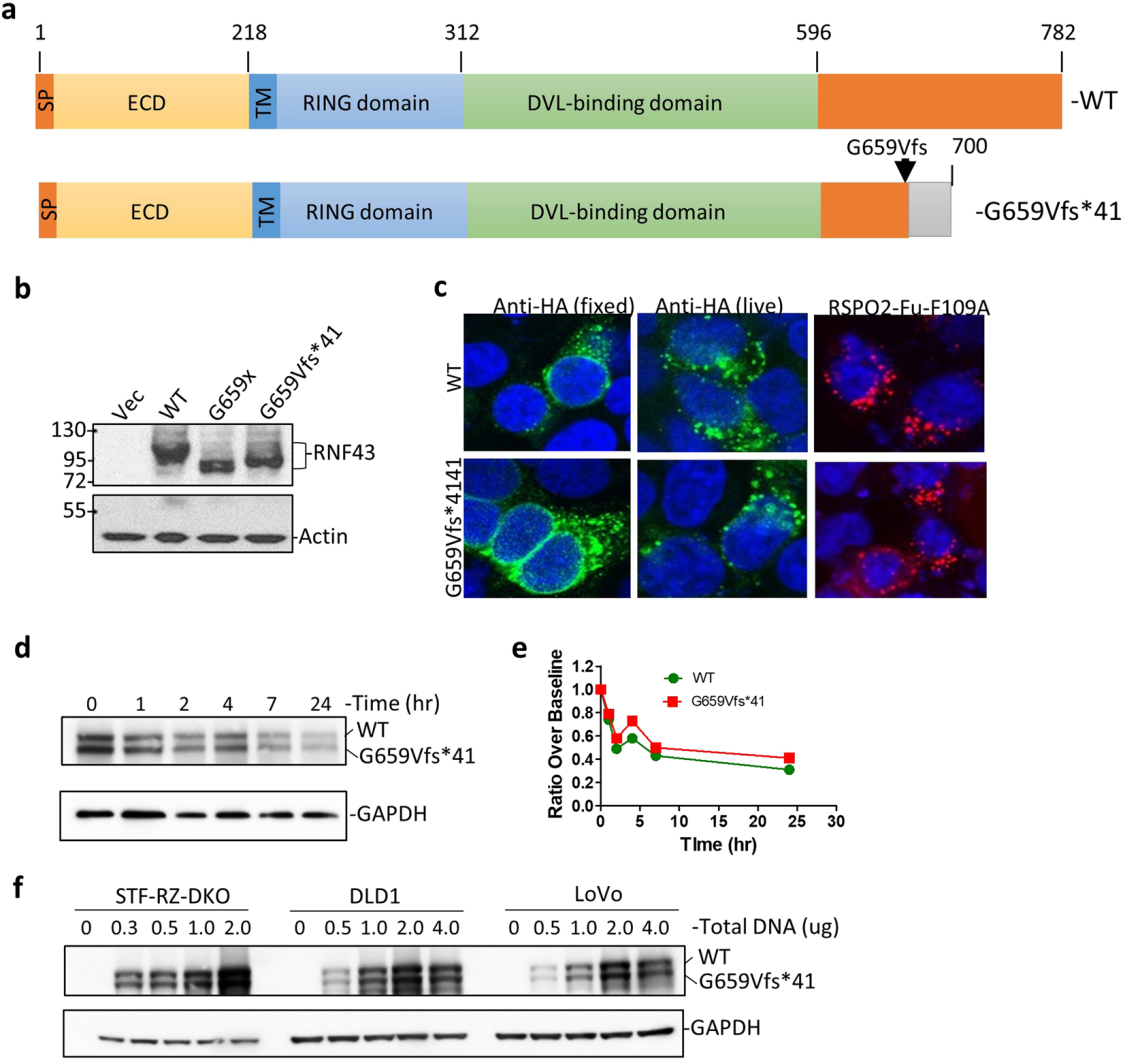
Expression, localization, RSPO binding, and stability of RNF43-G659Vfs*41 is similar to RNF43-WT. (**a**) Schematic diagram of domain structures of RNF43-WT and RNF43-G659Vfs*41. ECD, extracellular domain; TM, transmembrane domain; RING, RING E3 ligase domain. (**b**) WB of HEK293T cells transfected with vector control (Vec), or HA-tagged RNF43-WT, -G659x, or -G659Vfs*41 using anti-HA antibody. (**c**) Confocal immunofluorescence microscopy of STF-RZ-DKO cells transfected with RNF43-WT and -G659Vfs*41 and stained with mouse anti-HA and Alexa488-labeled anti-mouse IgG (green), or incubated with RSPO2-Fu-F109A-Fc followed by staining with Alexa-555-labeled anti-human IgG (red). Nuclei were counter-stained with TO-PRO-3 Iodide (blue). (**d**) Anti-HA WB of STK-RZ-DKO cells co-transfected with RNF43-WT and –G659Vfs*41 at 1:1 ratio and treated with cycloheximide for the indicated periods of time. (**e**) Quantification of RNF43 in WB of (**d**) normalized over GAPDH. (**f**) Anti-HA WB of STF-RZ-DKO, DLD, and LoVo cells transfected with increasing amounts of DNA of HA-tagged RNF43-WT and G659Vfs*41 plasmids pre-mixed at 1:1 ratio. GAPDH and actin were loading controls.

To examine functional consequences of the G659Vfs*41 mutation, we generated constructs expressing the mature forms of full-length wild type (WT) RNF43 or G659Vfs*41 with CD8 signal peptide followed by an HA tag at the N-terminus (Fig. 2a). An additional construct with a termination codon immediately following G659 (G659x) was also generated to determine if the 41-AA neopolypeptide affects expression. The three constructs were transfected into HEK293T cells and Western blot (WB) analysis showed that they were expressed at similar levels with the expected differences in molecular weights (Fig. 2b), indicating that a single G-C bp deletion between bp1969–1976 of the RNF43 open reading frame would indeed shift the frame and extend the length of the protein beyond G659. Subsequent work only used the G659Vfs*41 construct as this carried the actual cancer mutation.

To examine functional consequences of the RNF43 mutation, we generated a cell line with double knockout (KO) of RNF43 and ZNRF3 from the Wnt/β-catenin signaling reporter cell line HEK293-STF cells using the CRISP/Cas9 method as previously described^5,23,24^. As expected, the double KO cells (STF-RZ-DKO) had higher response to Wnt stimulation with no further response to RSPO1 treatment (Supplementary Fig. S2a). HA-tagged RNF43-WT and –G659Vfs*41 were then transfected into STF-RZ-DKO cells and examined by immunofluorescence. In fixed then permeabilized cells, staining with anti-HA antibody showed that both WT and G659Vfs*41 were mostly found in intracellular vesicles with strong perinuclear localization (Fig. 2c, left panel). In live cell staining, anti-HA antibody was rapidly internalized into vesicles in both WT and G659Vfs*41-transfected cells (Fig. 2c, mid panel). To test if the mutant was able to bind to RSPO, we used the fusion protein RSPO2-Fu-F109A-IgG1-Fc which binds to ZNRF3/RNF43 with high affinity but does bind to LGR4 or LGR5^25^. In both WT- and G659Vfs*41-transfected cells, RSPO2-Fu-F109A showed strong binding and rapid internalization (Fig. 2c, right panel). To rule out potential of the non-native CD8 signal peptide on expression and ligand binding, we generated constructs expressing RNF43-WT and mutant with its native signal peptide and no other tag. Both WT and mutant showed strong binding of the ligand RSPO2-Fu-F109A with similar endocytic profile (Supplementary Fig. S3a). We then examined if the mutant had altered post-translational stability due to truncation of its native C-terminus and addition of the neopolypeptide. STF-RZ-DKO cells were co-transfected with HA-tagged RNF43-WT and G659Vfs*41 plasmids with a 1:1 ratio, and treated with cycloheximide for different periods of time. The level of both WT and G659Vfs*41 was nearly identical at 0 hr (Fig. 2d), and both WT and mutant decreased to a similar extent following the addition of cycloheximide (Fig. 2d,e), suggesting that two proteins had similar post-translational stability. Various amounts of WT and mutant RNF43 plasmids were also co-transfected at 1:1 ratio into two colon cancer cell lines (DLD1 and LoVo) as well as into STF-RZ-DKO cells, and WB analysis showed that the two proteins were detected at nearly identical levels across the range of expression (Fig. 2f). Overall, these results strongly suggest that G659Vfs*41 was similar to WT in expression, localization, ligand binding, and protein stability.

### RNF43-G659Vfs*41 is similar to RNF43-WT in inhibiting Wnt/β-catenin signaling and decreasing Wnt receptor level

**Figure 3:**
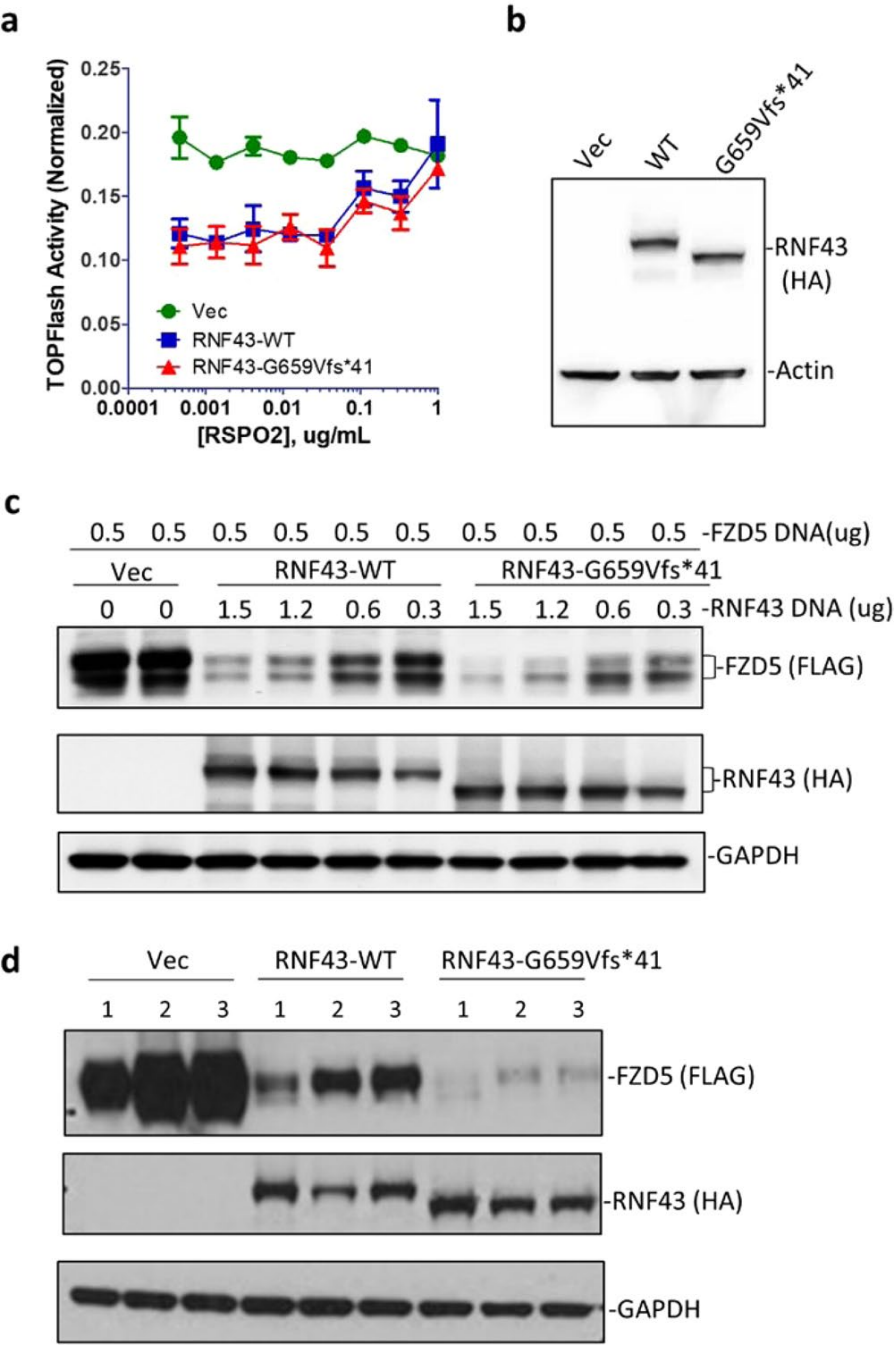
RNF43-G659Vfs*41 inhibited Wnt signaling and reduced FZD levels as well as RNF43-WT could. (**a**) TOPFlash activity of STK-RZ-DKO cells transfected with vector (Vec), RNF43-WT, or –G659Vfs*41 in response to RPSO2 treatment. (**b**) WB of cells from (**a**) with anti-HA antibody. (**c**) WB of FZD5 in STK-RZ-DKO cells transfected with vector, RNF43-WT, or –G659Vfs*41 with anti-FLAG and anti-HA antibody. (**d**) WB of FZD5 of HEK293T cells transfected with vector, RNF43-WT, or G659Vfs*41 and treated with vehicle or RSPO2-Furin-Fc proteins (R2Fu) at 10 µg/ml. 1 = vehicle; 2 = RSPO2Fu-WT; 3 = RSPO2Fu-F109A. Actin and GAPDP were loading controls.

We first compared functional activity of RNF43-WT and -G659Vfs*41 in the STF-RZ-DKO cells using the TOPFlash Wnt/β-catenin reporter enzyme assay^23^. Transfection of either WT or G659Vfs*41 led to approximately 50% decrease in reporter enzyme activity (Fig. 3a), and RSPO2 was able to restore the activity to the level of vector control cells in a dose-dependent manner (Fig. 3a) even though the potency of RSPO2 was much lower than in regular STF cells^25^. WB analysis of the cells showed both WT and mutant were expressed at similar levels (Fig. 3b). The incomplete suppression of Wnt activity in the TOPFlash assay (Fig. 3a) was likely due to only approximately half of the cells expressing RNF43 following transient transfection based on immunofluorescence. The diminished potency of RSPO2 was likely an effect of high recombinant expression of RNF43 protein. We also found that the activity of RNF43-WT and –G659Vfs*41 with its native signal peptide was similar to that of RNF43-WT with CD8 signal peptide in inhibiting Wnt signaling (Supplementary Fig. S3b). In addition, STF-RZ-DKO cells co-transfected with both WT and mutant RNF43 at 1:1 ratio of plasmid DNA showed a similar level of inhibition of Wnt signaling compared to RNF43-WT alone (Supplementary Fig. S3b), suggesting that the mutant had no dominant negative activity.

We then directly examined changes in the level of FZD protein following co-transfection of FLAG-tagged FZD5 and various amounts of WT or mutant RNF43 DNA into STF-RZ-DKO cells. WB analysis of FZD5 showed an approximate inverse correlation between levels of FZD5 and either RNF43-WT or G659Vfs*41 (Fig. 3c), consistent with the expected function of RNF43 which is to ubiqutinate FZD for degradation^2,3^. Furthermore, RSPO2-Fu-WT or –F109A were able to increase levels of FZD in HEK293T cells transfected with either RNF43-WT or –G659Vfs*41 to a similar extent (Fig. 3d), suggesting that mutant RNF43 was similar to WT in responding to RSPO with and without LGR. Intriguingly, in FZD degradation experiments, cells transfected with G659Vfs*41 appeared to have lower levels of FZD5 when compared to those in WT-transfected cells (Fig. 3c,d), suggesting that the mutant might be even slightly more active. Overall, these functional results clearly indicate that RNF43-G659Vfs*41 was fully capable of suppressing Wnt/β-catenin signaling and reducing FZD levels as much as RNF43-WT could.

### RNF43-G659Vfs*41 is associated with low Wnt signaling activity and BRAF-V600E mutation only in colon cancer

**Figure 4:**
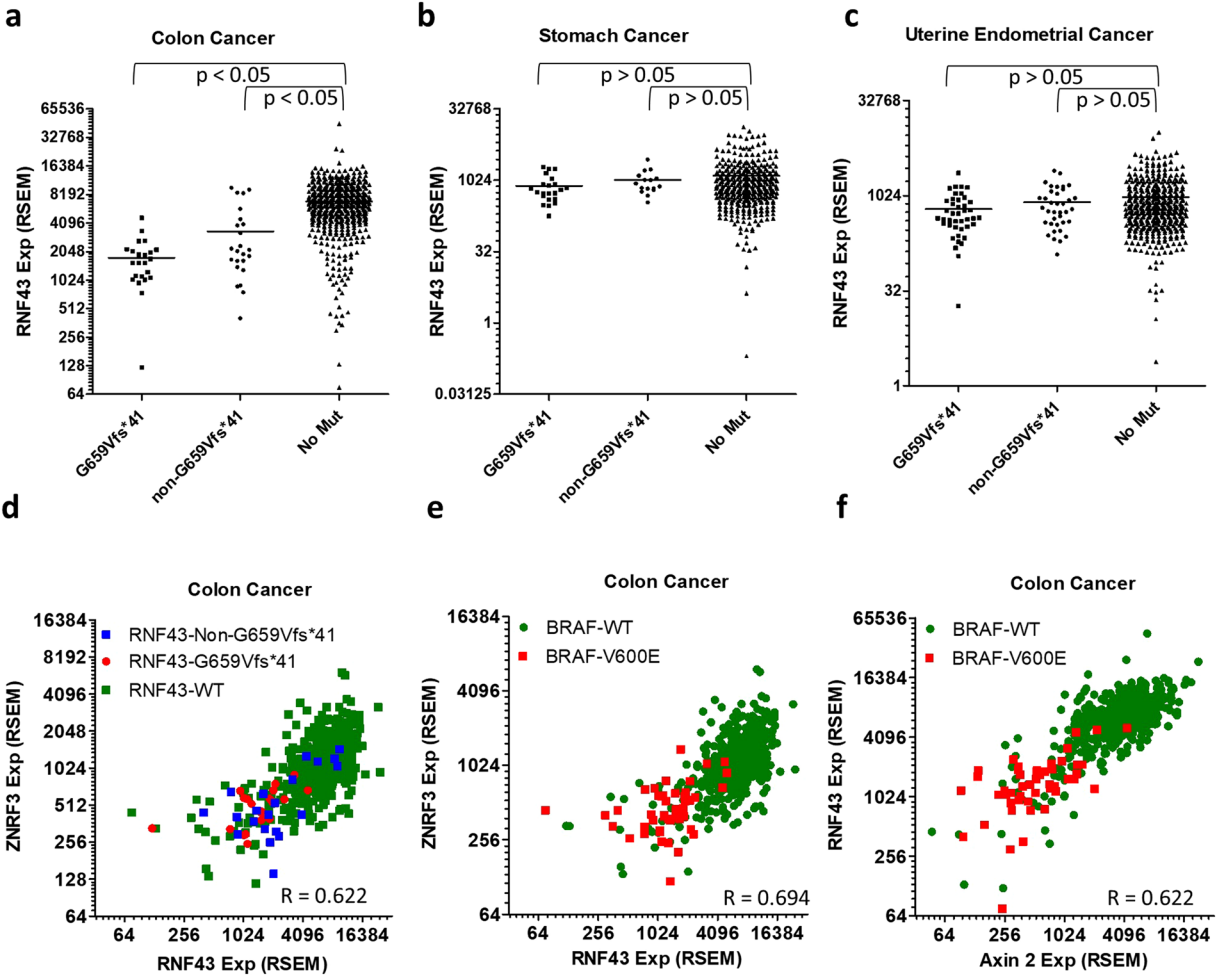
Expression of Axin2, RNF43, and ZNRF43 are highly correlated with each other and is low in RNF43-G659Vfs*41 and BRAF-V600E tumors only in colon cancer. (**a**) Scatter plot of expression level of RNF43 vs its own mutation status in colon cancer. (**b**) Expression level of RNF43 vs its own status in stomach cancer. (**c**) Expression level of RNF43 vs its own status in endometrial cancer. (**d**) Correlation between RNF43 and ZNRF3 in expression in relation to the mutation status of RNF43. (**e**) Correlation between RNF43 and Axin2 expression in relation to BRAF-V600E. (**f**) Correlation between RNF43 and ZNRF3 expression in relation to BRAF-V600E.

RNF43-G659Vfs*41 had been suggested to drive or facilitate colon cancer formation based on the presumption that it no longer functions in inhibiting Wnt signaling^7,9,14,26^. Since RNF43 is a target of Wnt signaling itself^2,3^, we examined the expression of RNF43 in relation to its own mutation status. Surprisingly, RNF43 expression in tumors with G659Vfs*41 mutation was much lower than in those without any mutation (mean RNA-seq RSEM value of 1746 vs 6897, p < 0.05, Fig. 4a). Tumors with non-G659Vfs*41 mutations in RNF43 also had lower expression of RNF43 (mean RNA-seq RSEM value = 3337, p < 0.05, Fig. 4a). However, in stomach and endometrial cancers, expression of RNF43 in G659Vfs*41 or non-G659Vfs*41 tumors was not different from those without any RNF43 mutation (Fig. 4b,c). Of note, expression of RNF43 in non-RNF43-mutated colon tumors was much higher than those in endometrial and stomach cancers (Fig. 4a–c). In fact, absolute mRNA levels of RNF43 in RNF43-G659Vfs*41colon cancer tumors was nearly identical to those in endometrial and stomach cancer (Fig. 4a–c). We then asked how ZNRF3 was related to RNF43 in expression and mutation status in colon cancer as both genes are targets as well as negative regulators of Wnt signaling. RNF43 and ZNRF3 expression were highly correlated (Spearman correlation efficient R = 0.622, Fig. 4d), consistent with both being Wnt signaling target genes. The data also implies that tumors with RNF43 mutations had low expression of both RNF43 and ZNRF3, an indicator of low Wnt signaling. If RNF43-G659Vfs*41 would lead to increase in Wnt signaling as APC mutations do, expression of RNF43 and ZNRF3 in RNF43-G659Vfs*14 tumors would follow the profile of tumors with APC mutation.

Previously, it was also noted that RNF43-G659Vfs*41 was frequently associated with BRAF-V600E mutation^9,14,26^, an expected correlation since BRAF-V600E is most frequent in MSI-H tumors with low MLH1 expression^27^. In the TCGA colon cancer cohort with 533 sequenced tumors, there are 21 tumors with G659Vfs*41 mutations and 47 tumors with BRAF-V600E mutation. Yet 14 of the 21 tumors with G659Vfs*41 mutation also had BRAF-V600E mutation (p = 0.0001, Fisher’s exact test), indicative of a strong association between the two mutations. As colon tumors with MSI-H and BRAF-V600E mutations were also known to have low Wnt signaling^27^, we analyzed expression levels of Axin2, a prototypical Wnt signaling target, and ZNRF3, relative to RNF43 and BRAF mutation status in the colon cancer cohort. Axin2 and ZNRF3 mRNA levels were highly correlated with that of RNF43 across all samples (overall Spearman’s correlation coefficient = 0.694 for Axin2, 0.622 for ZNRF3, Fig. 4e,f), consistent with all three being Wnt target genes in the intestinal tract. Remarkably, nearly all BRAF-V600E tumors had low expression of Axin2, RNF43, and ZNRF3 (Fig. 4e,f), but not vice versa, consistent with previous report of low Wnt signaling in colon tumors with MSI-H BRAF-V600E mutation^27^. Overall, these genomic data clearly indicate that a subset of colon cancers, including nearly of all those with BRAF-V600E mutation, had low Wnt signaling.

### Colon cancer cell line with RNF43-G659Vfs*41 and BRAF-V600E mutation is sensitive to activation of Wnt signaling

**Figure 5:**
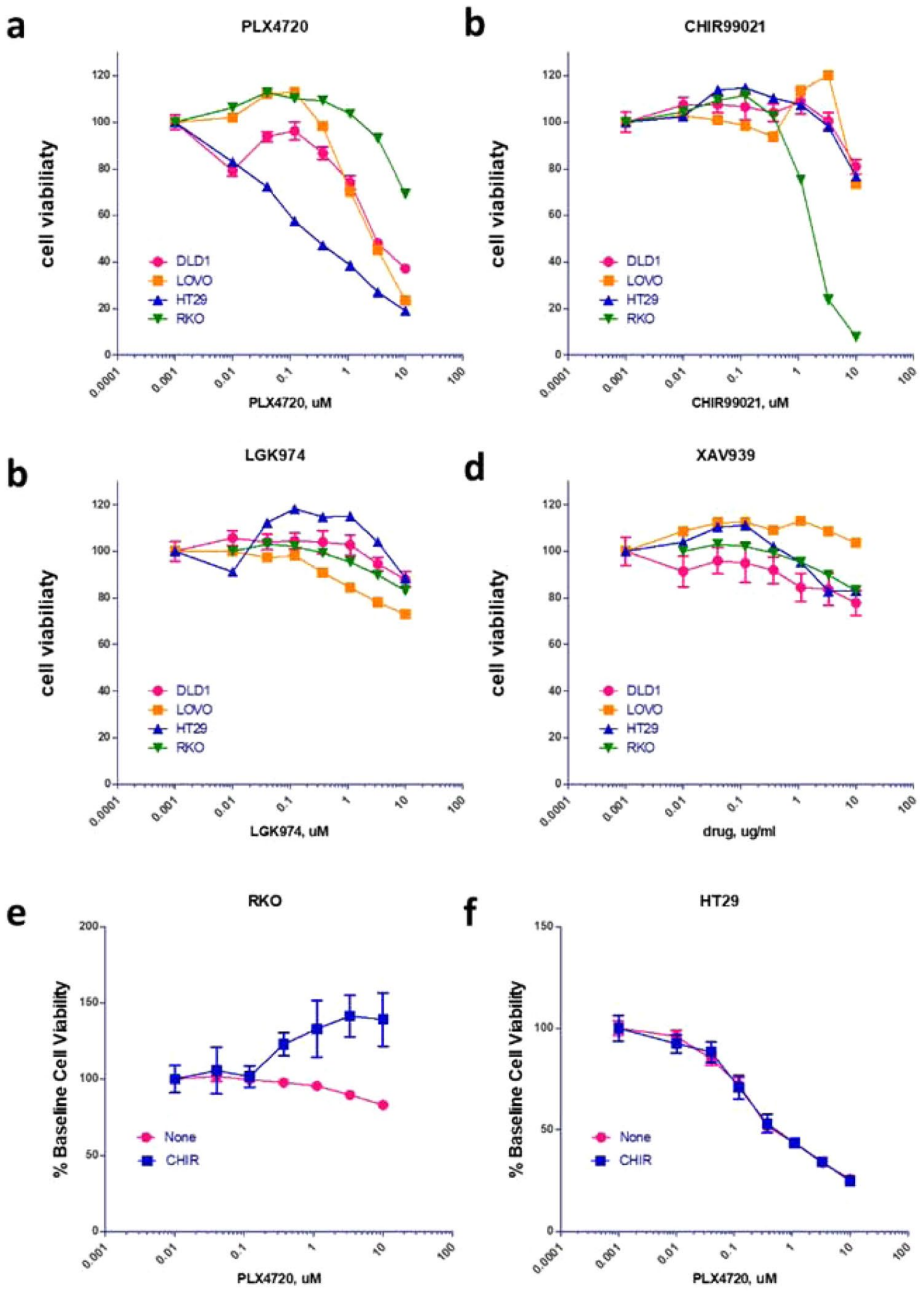
Cell viability of colon cancer cell line in response to treatment with inhibitors and activators of Wnt and BRAF signaling. (**a**) BRAF inhibitor PLX4720. (**b**) Wnt/β-catenin signaling activator CHIR99021. (**c**) Wnt ligand secretion inhibitor LGK974. (**d**) Wnt/β-catenin signaling inhibitor XAV939. (**e,f**) Combined effect of CHIR99021 and PLX4720 in RKO (**e**) and HT29 cells (**f**). For (**e**,**f**), varying concentrations of PLX4720 was tested together with vehicle or CHIR99021 at a fixed concentration of 3 uM.

As the majority of RNF43-G659Vfs*41 tumors had BRAF-V600E mutation and had low Wnt signaling, we mined the CCLE database for cell lines with these characteristics. The cell line RKO had the RNF43-G659Vfs*41 mutation as confirmed by Bond *et al*.^9^, BRAF-V600E and low Axin2 expression without APC mutation, making this cell line an apparent representative of the most common RNF43-G659Vfs*41 primary tumors. We also identified three other cancer cell lines with differential profiles in Wnt signaling gene expression and mutations as listed in Supplementary Table S1. Cell growth of the four cell lines were then tested side-by-side in the presence of various Wnt and BRAF signaling inhibitors: BRAF inhibitor PLX4720^28^, porcupine inhibitor LGK974 (inhibition of Wnt ligand secretion)^17^, tankyrase inhibitor XAV939 (stabilization of Axin1/2 and thus degradation of β-catenin)^29^, and GSK3 inhibitor CHIR99021 (inhibition of β-catenin phosphorylation and thus stabilization of β-catenin)^30^. Inhibition of BRAF (PLX4720) was highly effective in HT29 cells (BRAF-V600E, RNF43-WT, APC mutant) but had little activity in RKO cells (BRAF-V600E, RNF43-G659Vfs*41, APC-WT) (Fig. 5a), consistent with previously published results by others^31^. In LoVo and DLD1 cells (no RNF43 nor BRAF mutation), PLX4720 had intermediate effect (Fig. 5a). Remarkably, activation of Wnt signaling by the GSK3 inhibitor CHIR99021 was able to inhibit the growth of RKO cells completely with only minor effect on the other three cell lines at 10 µM (Fig. 5b). On the other hand, inhibition of Wnt signaling by either blockade of Wnt ligand synthesis (LGK974) or stabilization of Axin1 (XAV939) had little effect on the four cell lines (Fig. 5c,d) despite APC mutation in all cell lines except RKO. These data suggest that activation of Wnt signaling via inhibition of GSK3 was detrimental to RKO cells which have both RNF43-G659Vfs*41 and BRAF-V600E, suggesting that BRAF-V600E mutation was not compatible with high Wnt signaling under certain circumstances. This may be the underlying reason of why only MSI-H BRAF-V600E tumors had low Wnt signaling whereas BRAF-V600E tumors without MSI-H had high Wnt signaling^27^. Of note, HT29 cells are MSS (microsatellite stable) whereas RKO cells are MSI-H^32^.

If high Wnt signaling due to GSK3 inhibition is incompatible with high BRAF activity (BRAF-V600E), we would expect that inhibition of BRAF may rescue the cells from high Wnt signaling. To test this, we compared the effect of combining PLX4720 with CHIR99021 in RKO and HT29 cells. In RKO cells, PLX4720 had little effect by itself but was able to partially rescue the effect of CHIR99021 which inhibited cell growth by ~50% at the used concentration (3 uM) (Fig. 5e). In HT29 cells, CHIR99021 at the same concentration had no effect on PLX4720 (Fig. 5f). These data strongly suggest that in RKO cells, cell growth inhibition by CHIR99021 depended on high BRAF activity, consistent with the model that high Wnt signaling was not tolerated with BRAF-V600E in MSI-H tumors.

### GSK3 inhibition in RKO cells led to stabilization of β-catenin

**Figure 6:**
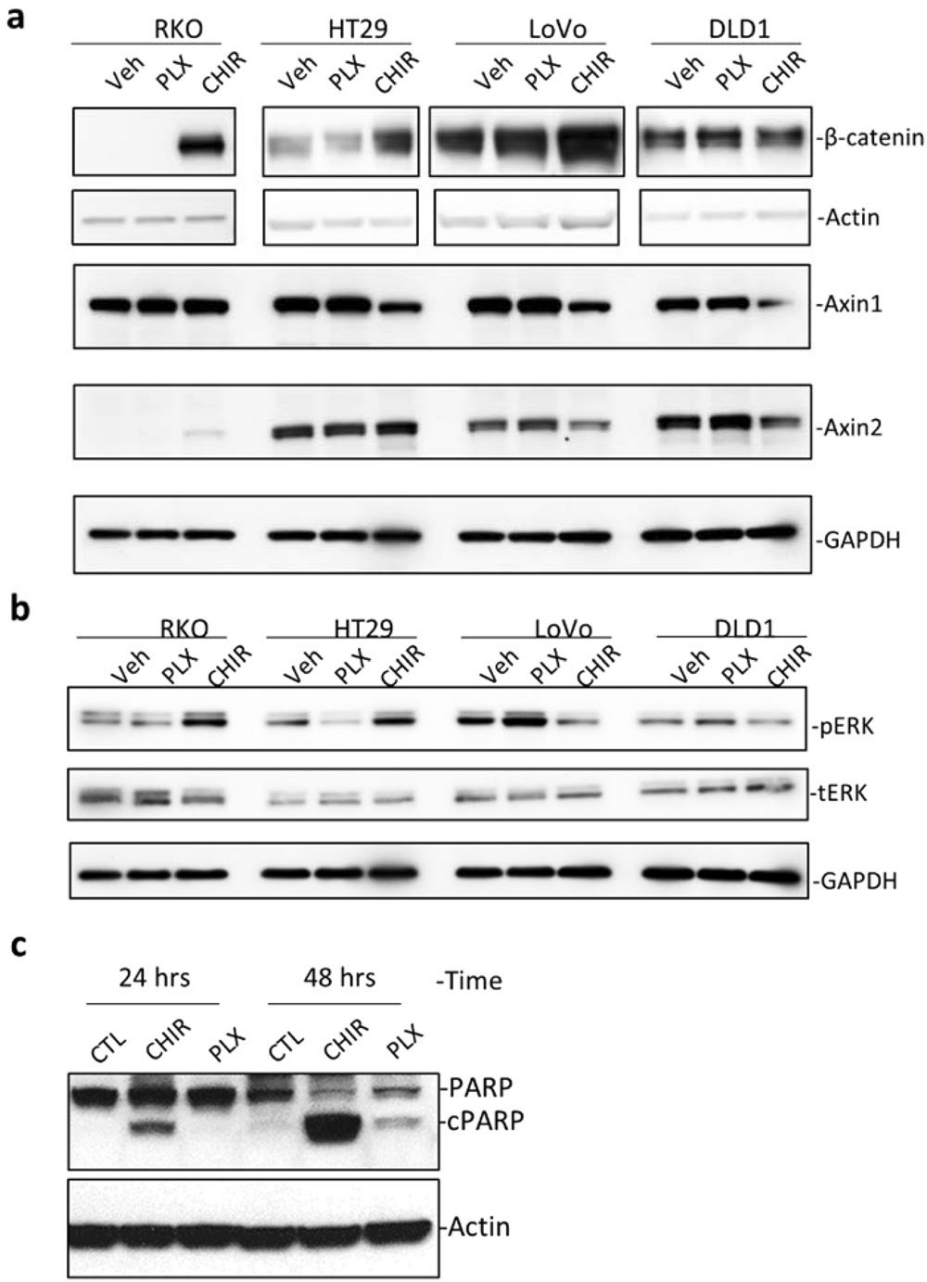
CHIR99021 induced apoptosis in RKO cells by stabilizing β-catenin. (**a**) WB analysis of Wnt signaling pathway indicators in the four cell lines treated with vehicle control (CTL), CHIR99021 (CHIR), or PLX4720, each at 3 µM for 24 hours. (**b**) WB analysis of phosphor-ERK (pERK) and total ERK (tERK) in the four cell lines treated with the indicated compounds at 3 µM for 24 hours. (**c**) WB of PARP1 cleavage in RKO cells following treatment with CHIR99021 or PLX4720 at 3 µM for the indicated period of time. cPARP1 = cleaved PARP1. Actin and GAPDH were loading controls.

To determine the mechanism of sensitivity of RKO cells to GSK3 inhibition, we first examined Wnt and MAPK signaling pathways in the four cell lines. RKO cells had little β-catenin in the cytoplasm at base line and CHIR99021 treatment increased its level as expected (Fig. 6a). CHIR99021 also increased the level of cytoplasmic β-catenin in HT29, and LoVo cells but not in DLD1 cells (Fig. 6a). Level of Axin2 was only increased in RKO cells with CHIR99021, probably because the other three cell lines are mutated in APC and thus had already saturated in Axin2 expression. Levels of Axin1 were decreased in all CHIR99021-treated cell lines except RKO, likely due to degradation of the β-catenin destruction complex as a result of lack of phosphorylated β-catenin. In contrast, PLX4720 had no effect on the markers of Wnt signaling pathway in the four cell lines (Fig. 6a). In the MAPK pathway, HT29 cells showed the best response in pERK reduction with PLX4720 treatment, consistent with its high sensitivity. RKO cells, despite having BRAF-V600E mutation, only had a minor reduction in pERK. LoVo cells showed an increase in pERK, most likely due to positive feedback following inhibition of BRAF in cells with KRAS mutation. We then asked if high Wnt signaling would induce apoptosis in RKO cells. As shown in Fig. 6c, a significant increase of PARP cleavage was observed in RKO cells treated with CHIR99021 but not PLX4720. Overall, these results confirmed that GSK3 inhibition in RKO cells resulted in dramatic increase in the level of cytoplasmic β-catenin and Axin2, which led to apoptosis and cell death.

## Discussion

RNF43 and its related homolog ZNRF3 are key negative regulators of Wnt signaling and both were found to be mutated in all major types of solid tumors at relatively low frequency. Some of the null or missense mutations of RNF43 located in the N-terminal half had been shown to be loss-of-function and presumably play a role in tumorigenesis of the pertinent tumors^4,17^. The most common recurrent mutation is RNF43-G659Vfs*41 which has been “automatically” assumed to be a loss-of-function mutation that would lead to increase in Wnt signaling and thus drive or facilitate tumorigenesis. Yet, no evidence has been presented to show that the mutation abolishes the enzyme function as a driving mechanism in oncogenesis. Here we show that this mutation itself does not inactivate RNF43 in suppressing Wnt signaling.

For both ZNRF3 and RNF43, three domain (ECD, RING E3 ligase domain, and DVL-binding domains) were shown to be essential for their activity in ubiquitinating FZD and inhibiting Wnt signaling^4,5^. RNF43- G659Vfs*41 replaces the C-terminal 124-AA region of RNF43 with a 41-AA neopeptide that shows no homology to any other protein or functional motif. The replaced region is far downstream of the DVL-binding domain and previous studies by others showed that deletion of the entire region downstream AA-596 (end of DVL-binding domain) had no effect on RNF43’s activity in inhibiting Wnt signaling^4,5^. In theory, it is possible that the 41-AA neopolypeptide could somehow inhibit the function of the enzyme and increase Wnt signaling. Here we excluded this possibility by demonstrating that the expression, localization, and function of recombinant RNF43 with the very G659Vfs*41 mutation were no different from those of RNF43-WT. In cancer cells, the mutant RNF43 would co-express with WT RNF43, and could possibly inhibit the function of the WT enzymes if WT and mutant enzymes could form inactive dimers. We showed that co-transfection of WT and G659Vfs*41 still inhibited Wnt signaling, indicating lack of dominant negative effect. These results clearly indicate that G659Vfs*41 is not a loss-of-function mutation. The question is whether the mutant protein was made at much reduced levels due to nonsense-mediated decay (NMD) of the mutant mRNA^33^. G659Vfs*41 was reported to elicit NMD in colon and stomach cancers though the extent of decay was predicted to be moderate^34^. Yet, Roudko *et al*. just reported that RNF43-G659Vfs*41 was detected as a neopolypeptide that was predicted to be a highly immunogenic tumor antigen in the MSI-H subtypes of the three tumor types (colon, stomach, and endometrial cancers)^35^. Since nearly all G659Vfs*41 tumors are still expected to co-express WT ZNRF3 and RNF43 (there was no loss of genomic copy number of RNF43, Supplementary Fig. S5), even reduced expression of G659Vfs*41 in affected tumors would still have little effect on Wnt signaling. In mouse models, intestinal organoids with homozygous G659Vfs*41 mutations could survive without RSPO only after double KO with ZNRF3^14^, indicating that G659Vfs*41 itself was insufficient to drive up Wnt signaling to support organoid growth. Of note, the RNF43 mutation generated by CRISPR in mouse organoids had various deletions in the region corresponding to the human 7-G repeat^14^, which may elicit different NMD than human G659Vfs*41. Together, these findings strongly suggest that RNF43-G659Vfs*41 is unlikely to be a driver of tumorigenesis.

Multiple studies reported the association of RNF43-G659Vfs*41 with BRAF-V600E in MSI-H colon tumors^9,12–14,26^. However, the allele frequency of RNF43 and BRAF mutations were not correlated (supplementary Fig. S3), suggesting that the events were independent. Also, in stomach and endometrial cancers in which BRAF-V600E was not found, RNF43-G659Vfs*41 occurred in a frequency similar to those of MSI-H colon cancer. In all three cancers types with high incidence of RNF43-G659Vfs*41, tumors with G659Vfs*41 all had low expression of MLH1 and presumably defective DNA repair, suggesting that replication of the seven G repeat in coding sequence of RNF43 is very prone to error and highly dependent on MLH1 for correction. Together with the finding that G659Vfs*41 had no effect on RNF43’s activity in Wnt signaling, these data strongly suggest that G659Vfs*41 is a passenger mutation that happens to occur at high frequency due to the lack of MLH1.

The association of BRAF-V600E and low Wnt signaling was only found in MSI-H colon tumors which were rarely mutated in APC^27^. In melanoma cells with BRAF-V600E mutation, inhibition of BRAF by PLX4720 led to cell death and was dependent on increased Wnt/β-catenin signaling as a result of decreased Axin1, a negative regulator of β-catenin^36^. In colon cancer cell lines with or without BRAF-V600E mutation, we found that PLX had no effect on the level of Axin1 nor β-catenin (Fig. 6A), indicating that BRAF-V600E had distinct effect in colon cancer and melanoma cells. In cell growth tests of the two colon cancer cell lines with BRAF-V600E mutation, HT29 (MSI-low, not hypermutated) and RKO (MSI-HI)^32^, HT29 cells were highly sensitive to PLX4720 whereas RKO cells were rather resistant which is consistent with previous report^31^. Instead, we found RKO cells was highly sensitive to a Wnt/β-catenin signaling activator CHIR-99021 which was not known to be cytotoxic to any other cell line. In melanoma cells with BRAF-V600E mutation, inhibition of BRAF by PLX was synergistic with activation of Wnt signaling in causing cell death^36^, suggesting that low BRAF activity is incompatible with high Wnt signaling. In RKO cells, however, PLX4720 would slightly counteract the effect of CHIR99021, suggesting that low BRAF activity is more compatible with high Wnt signaling. This would imply that high BRAF activity in BRAF-V600E cells would require low Wnt signaling, with the caveat that only one cell line was tested. Nevertheless, the finding is consistent with low levels of Axin2 and other Wnt target genes as well as the lack of APC mutation in MSI-H colon cancer cells with BRAF-V600E mutation^37^. Interestingly, in intestinal stem cells, high Wnt signaling and MAPK activity were shown to be incompatible as the two pathways seem to inhibit each other^36,38,39^. Taken together, these findings suggest that MSI-H tumors with BRAF-V600E mutation might have evolved to have low Wnt signaling due to incompatibility of hyperactive MAPK with high Wnt signaling.

In conclusion, we found that RNF43-G659Vfs*41 was able to inhibit Wnt signaling as much as the WT enzyme could and that this mutation was most likely a passenger mutation due to error-prone replication of a seven G repeat in its open reading frame in MLH1-deficient tumor cells. Its coexistence with BRAF-V600E was most likely a pure coincidence for both being enriched in MSI-H tumors. A colon cancer cell line with RNF43-G659Vfs*41 and BRAF-V600E mutations had low levels of Wnt signaling and was inhibited by activation of Wnt signaling. Further studies will be needed to test the possibility of MSI-H colon cancers with BRAF-V600E being sensitive to Wnt activation.

## Materials and Methods

### Plasmids and cloning

Plasmids encoding N-terminal HA-tagged RNF43-WT and -G659x with a CD8 signal peptide were cloned into pIRESpuro3 by standard PCR and In-Fusion HD cloning kit (Clonetech). HA-RNF43-G659Vfs*41 was then generated by deleting a G in bp 1979–1976 of the RNF43 open reading frame in the HA-RNF43-WT plasmid by site-directed mutagenesis. WT and G659Vfs*41 plasmids with its native signal peptide were generated by replacing the CD8 signaling peptide and HA tag with its native signal peptide sequence by In-Fusion® cloning. FLAG-tagged FZD5 was cloned into pIRESpuro3 as described previously^25^. All plasmids were verified by complete DNA sequencing.

### Recombinant proteins, antibodies, and western blotting

Recombinant full-length human RSPO2 was purchased from R&D Systems. RSPO2-Fu-WT and RSPO2-Fu-F109A were purified as described previously^25^. For western blot analysis, anti-HA (Invitrogen cat #71–5500), anti-FLAG (Sigma cat # F7425), anti-β-actin (Cell Signaling cat #4970), anti-β-catenin (Cell Signaling cat #9562), anti-Axin1 (Cell Signaling cat #2087), anti-Axin2 (Cell Signaling cat #2151), anti-phosphor-ERK (Cell Signaling cat #9101), total ERK (Cell Signaling cat #4695), anti-PARP (cell signaling cat #9532), and anti-GAPDH (Cell Signaling cat #2118) were used. For ICC, anti-HA-Alexa 488 (Cell Signaling cat #2350), anti-human-Alexa 488 or –Alexa 555 (Invitrogen cat #A11013 and #A21433) were used. For western blotting, cells were lysed with RIPA buffer (50 mM Tris-Cl pH 7.4, 150 mM NaCl, 1 mM DTT, 1% Triton X-100, 1% sodium deoxycholate, 0.1% SDS), supplemented with protease and phosphatase inhibitors, and reduced at 37 °C for 1 hr. HRP-conjugated secondary rabbit or mouse antibodies (Cell Signaling) were used following the standard ECL protocol. For cytoplasmic β-catenin analysis, whole cell lysates were incubated with agarose bound concanavalin A beads to remove membrane-bound β-catenin and the remaining fractions were used as previously described^40^.

### Cell culture, transient transfection, and protein stability analysis

All cell lines were purchased from the ATCC and cultured in a 37 °C humidified incubator containing 5% CO_2_. HEK293T, HEK293-STF and HT29 cells were cultured in DMEM, DLD1 and Lovo cells in RPMI-1640, and RKO cells in MEM media. All media were supplemented with 10% FBS, 100 units/mL penicillin, and 100 mg/mL streptomycin. For transient transfection of HEK293 and STF-derived cells, ~80% confluent cells were transfected with DNA: FuGENE HD (Promega) ratio of 1:3 for all transient transfection presented. Transfection of DLD1 and LoVo cells were performed with jetPEI® (Polyplus-Transfection). For the protein stability analysis, cells with treated with cycloheximide at 100 µg/ml for the indicated periods of time and harvested, followed by WB analysis.

### Generation of STF RNF43 and ZNRF3 double knockout cells

For CRISPR-based knockout of ZNRF and RNF43 in HEK293-STF cells, the guide sequences GCAGGGTAGCCATCAGCAGCC (corresponding to nucleotide 44–64 of human RNF43 open reading frame) and AGGACTTGTATGAATATGGC of ZNRF3 (corresponding to nucleotide 338–357 of human ZNRF3 open reading frame) were cloned into the vector Lenti-CRISPR2 as described^24^. The two guide sequences were provided by Dr. Feng Cong as they were used in the generation of double knockout of RNF43 and ZNRF in HEK293 cells^5^. Lentiviral particles of lenti-CRISPR2 containing RNF43 and ZNRF3 guide sequence were used to co-infect HEK293-STF cells and cells were selected with puromycin at 1 µg/ml. Single colonies were isolated and analyzed for Wnt and RSPO response by the TOPFlash assay^23^. One clone (D8) showed complete loss of response to RSPO1 in the TOPFlash assay (supplementary Fig. S1a) and knockout of both RNF43 and ZNRF3 in D8 cells were verified by sequencing the genomic regions flanking the two guide sequences. In brief, genomic sequences were amplified by PCR using forward primers CGAAGTGACATTCAATCACAAG and GACGTTACTTTTGGCTATAGCATCTG (located in intron 1 of RNF43 and ZNRF3, respectively) and reverse primers CCTTCTGCTGGAGTTATTTCAGC and CGACAAGAGGGTAGAGCCCGCTC (located in intron 2 of RNF43 and ZNRF3, respectively). The PCR products were cloned into pCR2.1 vector using TA cloning kit (Invitrogen) and a total of 16 clones were sequenced with the identified mutations shown in Supplementary Fig. S1b.

### Wnt/β-catenin signaling TOPFlash reporter enzyme assay and immunofluorescence

TOPFlash assay in STF RNF43-ZNRF3 double KO (RZ-DKO) cells transfected with vector, RNF43-WT, or RNF43-G659Vfs*41 were carried out as described previously^25^. All TOPFlash experiments were repeated at least three times with duplicates or triplicates in each experiment. For RNF43 localization, STF-RZ-DKO cells transfected with RNF43-WT or G659Vfs*41 were treated as indicated. The treated cells were incubated at 37 °C with 95% humidity and 5% CO_2_ for 1 hr and fixed with 4.2% paraformaldehyde, followed by permeabilization with 0.1% saponin. Then, the secondary antibody, anti-human-Alexa 555 was used to label Fc-tagged RSPO2-Fu. Cells were imaged under the confocal microscope and analyzed by the Leica LAS AF Lite software.

### *In vitro* cell viability assays

Cells were seeded at various numbers (depending on growth rate) in half-area white 96-well plates and serial dilutions of the compounds were added. The cells were incubated for 4 days and cell viabilty was measured using CellTiter-Glo® Luminescent Cell Viability Assay (Promega).

### Cancer genomics data and statistical analysis

All expression and mutation data of TCGA tumor samples were doanloaded from cBioportal^20,21^. Data plotting and statistical analysis were carried out using GrpahPad Prism.

## Supplementary information

Supplementary Figures and original gel files

## Data Availability

All data generated or analyzed in this study are included in this published article and its Supplementary Information Files.

## References

[1] Yagyu R (2004). A novel oncoprotein RNF43 functions in an autocrine manner in colorectal cancer. International journal of oncology.

[2] Hao HX (2012). ZNRF3 promotes Wnt receptor turnover in an R-spondin-sensitive manner. Nature.

[3] Koo BK (2012). Tumour suppressor RNF43 is a stem-cell E3 ligase that induces endocytosis of Wnt receptors. Nature.

[4] Tsukiyama Tadasuke, Fukui Akimasa, Terai Sayuri, Fujioka Yoichiro, Shinada Keisuke, Takahashi Hidehisa, Yamaguchi Terry P., Ohba Yusuke, Hatakeyama Shigetsugu (2015). Molecular Role of RNF43 in Canonical and Noncanonical Wnt Signaling. Molecular and Cellular Biology.

[5] Jiang X, Charlat O, Zamponi R, Yang Y, Cong F (2015). Dishevelled Promotes Wnt Receptor Degradation through Recruitment of ZNRF3/RNF43 E3 Ubiquitin Ligases. Mol Cell.

[6] Wu J (2011). Whole-exome sequencing of neoplastic cysts of the pancreas reveals recurrent mutations in components of ubiquitin-dependent pathways. Proc Natl Acad Sci USA.

[7] Giannakis M (2014). RNF43 is frequently mutated in colorectal and endometrial cancers. Nat Genet.

[8] Wang K (2014). Whole-genome sequencing and comprehensive molecular profiling identify new driver mutations in gastric cancer. Nat Genet.

[9] Bond CE (2016). RNF43 and ZNRF3 are commonly altered in serrated pathway colorectal tumorigenesis. Oncotarget.

[10] Hashimoto T (2017). WNT Pathway Gene Mutations Are Associated With the Presence of Dysplasia in Colorectal Sessile Serrated Adenoma/Polyps. The American journal of surgical pathology.

[11] Jo YS (2015). Frequent frameshift mutations in 2 mononucleotide repeats of RNF43 gene and its regional heterogeneity in gastric and colorectal cancers. Human pathology.

[12] Sekine S (2016). Frequent PTPRK-RSPO3 fusions and RNF43 mutations in colorectal traditional serrated adenoma. J Pathol.

[13] Tsai JH (2016). RNF43 Is an Early and Specific Mutated Gene in the Serrated Pathway, With Increased Frequency in Traditional Serrated Adenoma and Its Associated Malignancy. The American journal of surgical pathology.

[14] Yan HHN (2017). RNF43 germline and somatic mutation in serrated neoplasia pathway and its association with BRAF mutation. Gut.

[15] Tate JG (2019). COSMIC: the Catalogue Of Somatic Mutations In Cancer. Nucleic Acids Res.

[16] Neumeyer Victoria, Vieth Michael, Gerhard Markus, Mejías-Luque Raquel (2019). Mutated Rnf43 Aggravates Helicobacter Pylori-Induced Gastric Pathology. Cancers.

[17] Jiang X (2013). Inactivating mutations of RNF43 confer Wnt dependency in pancreatic ductal adenocarcinoma. Proc Natl Acad Sci USA.

[18] Seshagiri S (2012). Recurrent R-spondin fusions in colon cancer. Nature.

[19] Gong X (2015). Aberrant RSPO3-LGR4 signaling in Keap1-deficient lung adenocarcinomas promotes tumor aggressiveness. Oncogene.

[20] Cerami E (2012). The cBio cancer genomics portal: an open platform for exploring multidimensional cancer genomics data. Cancer discovery.

[21] Gao J (2013). Integrative analysis of complex cancer genomics and clinical profiles using the cBioPortal. Sci Signal.

[22] Vilar E, Gruber SB (2010). Microsatellite instability in colorectal cancer-the stable evidence. Nature reviews. Clinical oncology.

[23] Xu Q (2004). Vascular development in the retina and inner ear: control by Norrin and Frizzled-4, a high-affinity ligand-receptor pair. Cell.

[24] Sanjana NE, Shalem O, Zhang F (2014). Improved vectors and genome-wide libraries for CRISPR screening. Nat Methods.

[25] Park S (2018). Differential activities and mechanisms of the four R-spondins in potentiating Wnt/beta-catenin signaling. J Biol Chem.

[26] Lai Chong, Sun Wenjie, Wang Xiaosheng, Xu Xingyu, Li Mengyuan, Huang Dongdong, Xu Enping, Lai Maode, Zhang Honghe (2019). RNF43 frameshift mutations contribute to tumourigenesis in right-sided colon cancer. Pathology - Research and Practice.

[27] Donehower LA (2013). MLH1-silenced and non-silenced subgroups of hypermutated colorectal carcinomas have distinct mutational landscapes. J Pathol.

[28] Tsai J (2008). Discovery of a selective inhibitor of oncogenic B-Raf kinase with potent antimelanoma activity. Proc Natl Acad Sci USA.

[29] Huang SM (2009). Tankyrase inhibition stabilizes axin and antagonizes Wnt signalling. Nature.

[30] Bennett CN (2002). Regulation of Wnt signaling during adipogenesis. J Biol Chem.

[31] Chen G (2018). Wnt/beta-Catenin Pathway Activation Mediates Adaptive Resistance to BRAF Inhibition in Colorectal Cancer. Mol Cancer Ther.

[32] Mouradov D (2014). Colorectal cancer cell lines are representative models of the main molecular subtypes of primary cancer. Cancer Res.

[33] Brogna S, Wen J (2009). Nonsense-mediated mRNA decay (NMD) mechanisms. Nature structural & molecular biology.

[34] Hu Z, Yau C, Ahmed AA (2017). A pan-cancer genome-wide analysis reveals tumour dependencies by induction of nonsense-mediated decay. Nature communications.

[35] Roudko, V. *et al*. Widespread immunogenic poly-epitope frameshift mutations in microsatellite unstable tumors. *bioRxiv*, 662262 (2019). 10.1016/j.cell.2020.11.004 PMC8025604 33259803

[36] Biechele TL (2012). Wnt/beta-catenin signaling and AXIN1 regulate apoptosis triggered by inhibition of the mutant kinase BRAFV600E in human melanoma. Sci Signal.

[37] Ryland GL (2013). RNF43 is a tumour suppressor gene mutated in mucinous tumours of the ovary. J Pathol.

[38] Kabiri Z (2018). Wnt signaling suppresses MAPK-driven proliferation of intestinal stem cells. J Clin Invest.

[39] Zhan T (2019). MEK inhibitors activate Wnt signalling and induce stem cell plasticity in colorectal cancer. Nature communications.

[40] Carmon KS, Gong X, Lin Q, Thomas A, Liu Q (2011). R-spondins function as ligands of the orphan receptors LGR4 and LGR5 to regulate Wnt/beta-catenin signaling. Proc Natl Acad Sci USA.

