# Supplementary material for: The most common RNF43 mutant G659Vfs*41 is fully functional in inhibiting Wnt signaling and unlikely to play a role in tumorigenesis
Source: Sci Rep. 2019 Dec 6;9:18557. doi: 10.1038/s41598-019-54931-3 (PMC6898356; doi:10.1038/s41598-019-54931-3)
Supplement: Supplementary file 1 — Supplementary Figures and original gel files [file 41598_2019_54931_MOESM1_ESM.pdf]

The most common RNF43 mutant G659Vfs*41 is fully functional in inhibiting Wnt signaling and unlikely to play a role in tumorigenesis

Jianghua Tu[1], Soohyun Park[1], Wangsheng Yu[1], Sheng Zhang[1], Ling Wu[1], Kendra Carmon[1] and Qingyun J. Liu[1]*

Supplementary Tables and Figures

Supplementary Table S1. Expression and mutation profile of 4 colon cancer cell lines

Cell line	Expression			Mutation				
	AXIN2	RNF43	ZNRF3	RNF43	APC	BRAF	KRAS	CTNNB1
HT29	6.3	9.9	10.3	None	T1556Nfs*3, E853*	V600E,T119S	None	None
RKO	4.7	5.0	6.6	X659fs-hom	None	V600E	None	None
DLD1	8.6	9.6	9.8	X659fs , L214M	S1426x	None	None	None
LOVO	7.3	7.6	8.5	None	R1114*, R2816Q, M1431Cfs*42	None	G13D	None

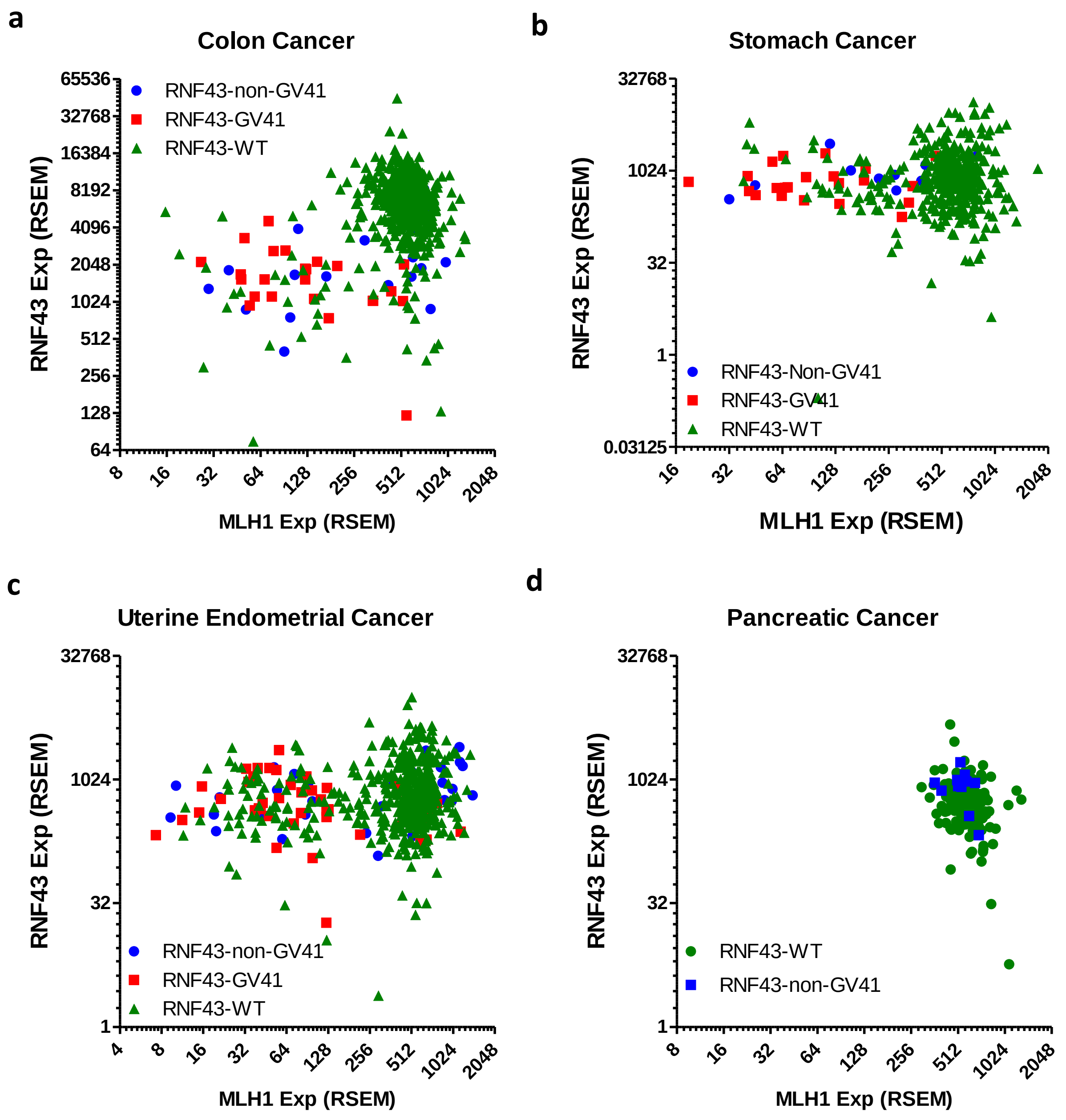

Supplementary Figure S1. RNF43-G659Vfs*41 mutations in colon, stomach, and endometrial cancers occurred nearly exclusively in tumors with low MLH1 expression. a-d, RNA-seq expression data and mutation status of RNF43 were plotted against RNA-seq data of MLH1 in colon (**a**), stomach (**b**), uterine endometrial (**c**), and pancreatic cancer (**d**). GV41 = G659Vfs*41.

a

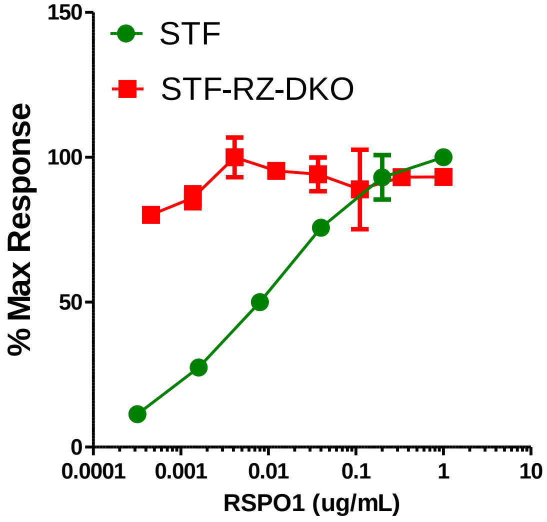

b

	Sequences of ZNRF3 Exon 2 and RNF43 Exon 2 in the STF clone D8 that had knockout of both RNF43 and ZNRF3.	# nucleotides changed
ZNRF3	5′- CGAAGAGGACTTGTATGAATATGGCTGGGTAGGAGTGGTGAAGCT -3′	
D8-1	5′- CGAAGAGGACTTGTATGAATAT**T**GGCTGGGTAGGAGTGGTGAAGCT -3′	+1
D8-2	5′- CGAAGAGGACTTG---------GGCTGGGTAGGAGTGGTGAAGCT -3′	-9
RNF43	5′- TGCCTGCAGGGTAGCCATCAGCAGCCAGGGCCAGAGGGCAGCCAGCTG -3′	
D8-1	5′- TGCCTGCA-GGTAGCCATCAGCAGCCAGGGCCAGAGGGCAGCCAGCTG -3′	-1
D8-2	5′- TGCC----------------------------AGAGGGCAGCCAGCTG -3′	-28

Supplementary Figure S2. Characterization of STF cells with double knockout of RNF43 and ZNRF3 (STF-RZ-DKO). (**a**) TOPFlash Wnt signaling assay results of parental STF and STF-RZ-DKO cells in response to RSPO1. (**b**) Sequence alignment of exon2 of RNF43 and ZNRF3 in STF cells with double knockout of the two genes. For both genes, the sequences of each allele are aligned with that of the WT sequence with the deleted sequences shown as gaps.

a

RNF43-WT RNF43-G659Vfs*41

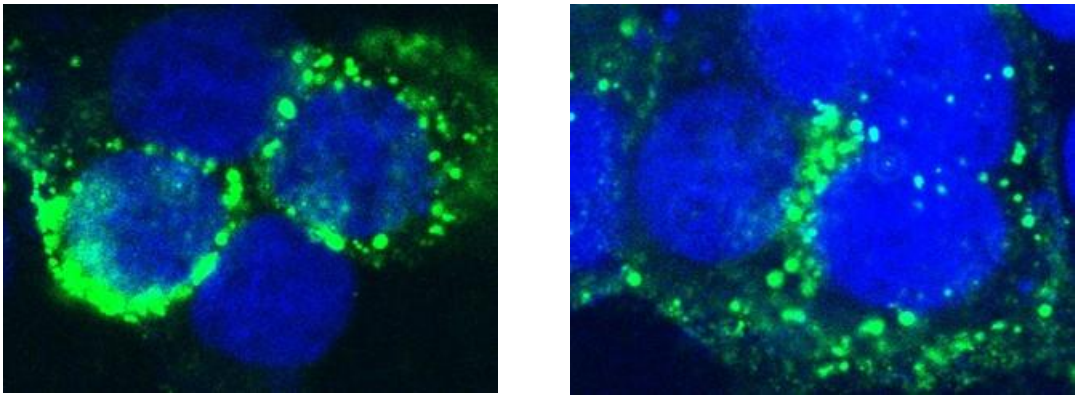

b

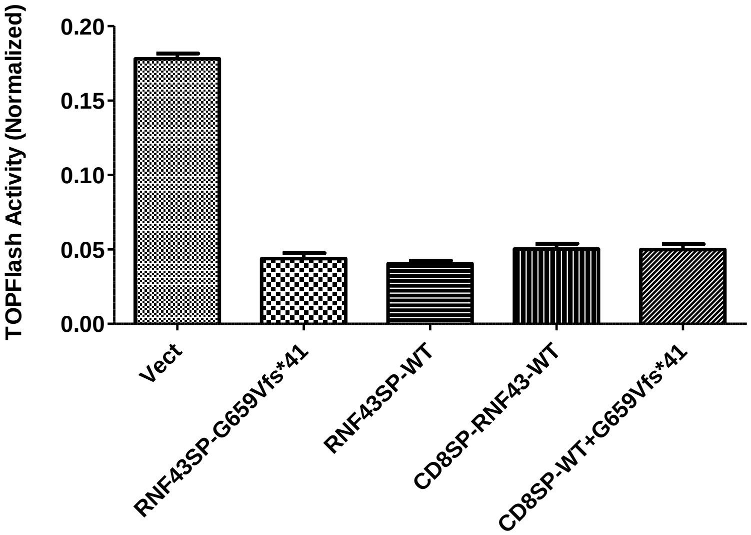

Supplementary Figure S3. RNF43-WT and –G659Vfs*41 with native signal peptide were nearly identical in binding and internalizing RSPO, as well as inhibiting Wnt signaling. (**a**) Confocal immunofluorescence microscopy of STF-RZ-DKO cells transfected with RNF43-WT or G659Vfs41 constructs that contain native signal peptide sequences. The cells were incubated with RSPO2-Fu-F109A-IgG1-Fc at 37 ^{o}C for one hour and then washed, fixed, permeabilized, followed by staining with Alexa488-labeled anti-human IgG (green). Nuclei were stained with TO-PRO-3 (blue). (**b**) TOPFlash results of STF-RZ-DKO cells transfected with the indicated plasmids following Wnt ligand stimulation. RNF43SP-G659Vfs*41 and –WT are mutant and wild-type RNF43 with its native signal peptide sequence, respectively. CD8SP-RNF43-WT is wild-type RNF43 with CD8 signal peptide and HA tag. The last column represents results of cells transfected with equal amounts of CD8SP-WT and CD8SP-G659Vfs*41 plasmids.

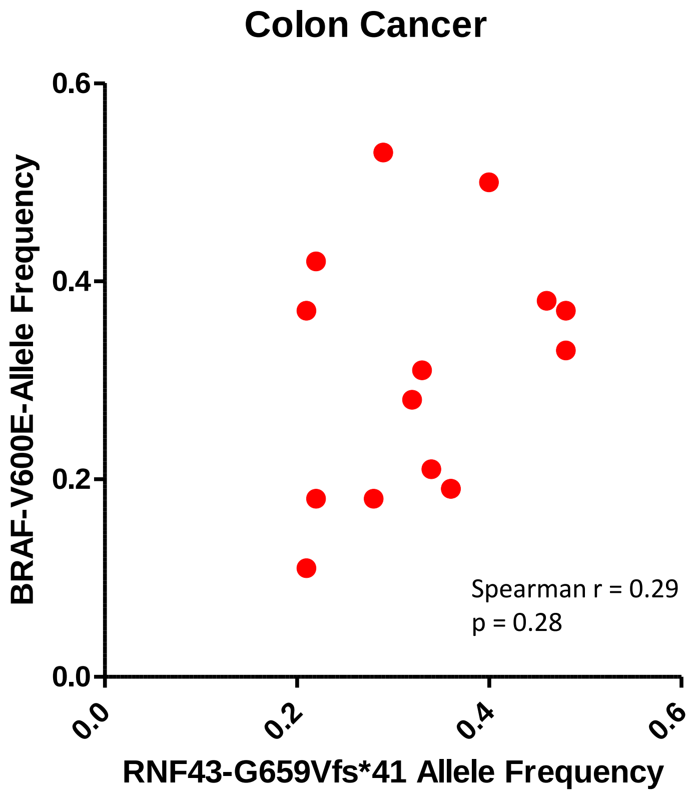

Supplementary Figure S4. No correlation between allele frequency of RNF43-G659Vfs*41 and BRAF-V600E in TCGA's colon cancer cohort. Of the 15 tumors with both RNF43-G659Vfs*41 and BRAF-V600E mutations, the respective allele frequencies were plotted as labeled.

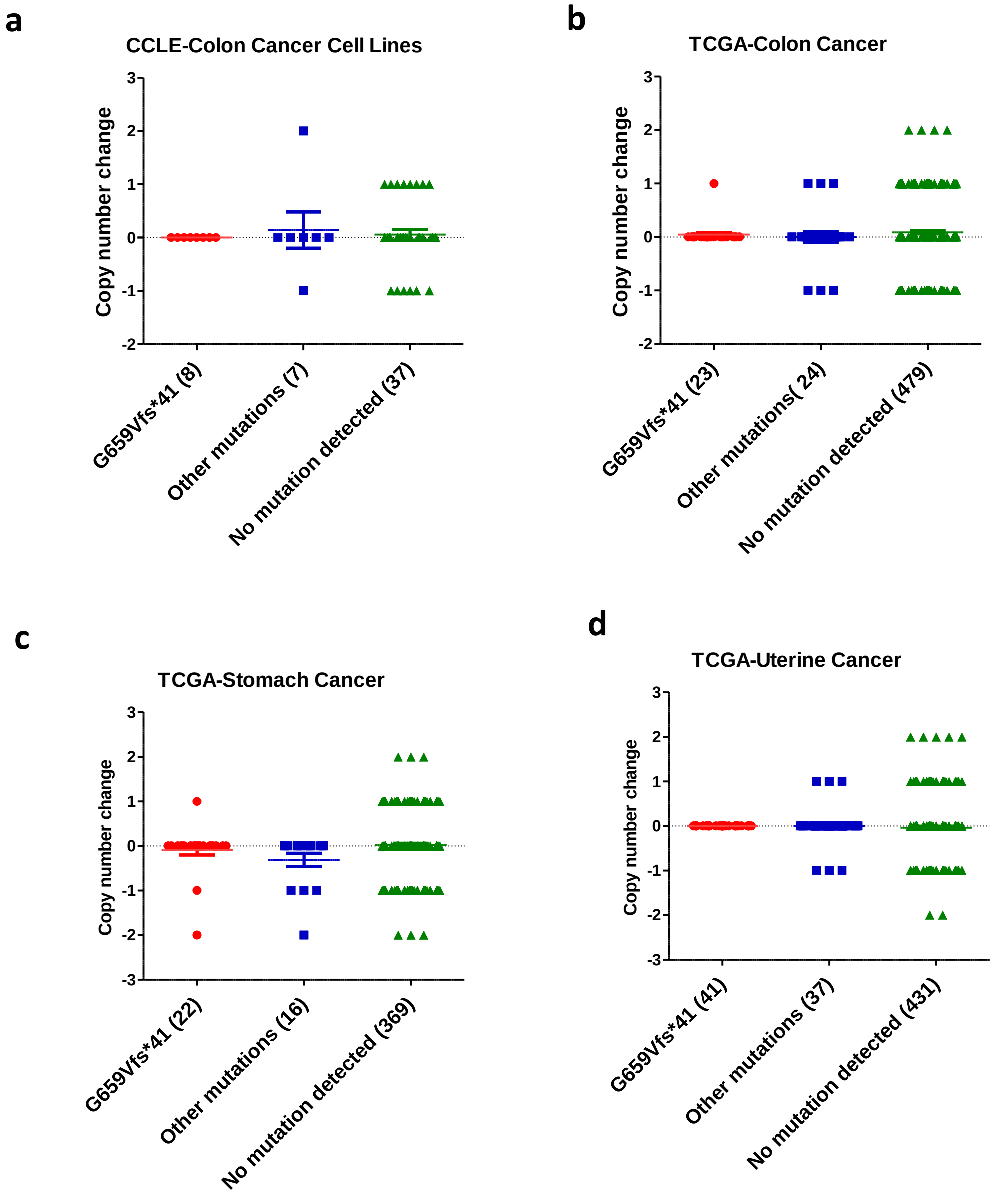

Supplementary Figure S5. No genomic copy number loss of RNF43 was detected in RNF43-mutated colon cancer cell lines and primary tumors. (**a**), Copy number change of RNF43 vs its mutation status in 52 colon cancer cell lines analyzed at CCLE. (**b-d**), Copy number change of RNF43 vs its mutation status in colon (**b**), stomach (**c**), and uterine cancer (**d**) in TCGA samples. The numbers in the parenthesis refer to the number of cell lines/cases for each genotype. Other mutations denote RNF43 mutations that are not G659Vfs*41.

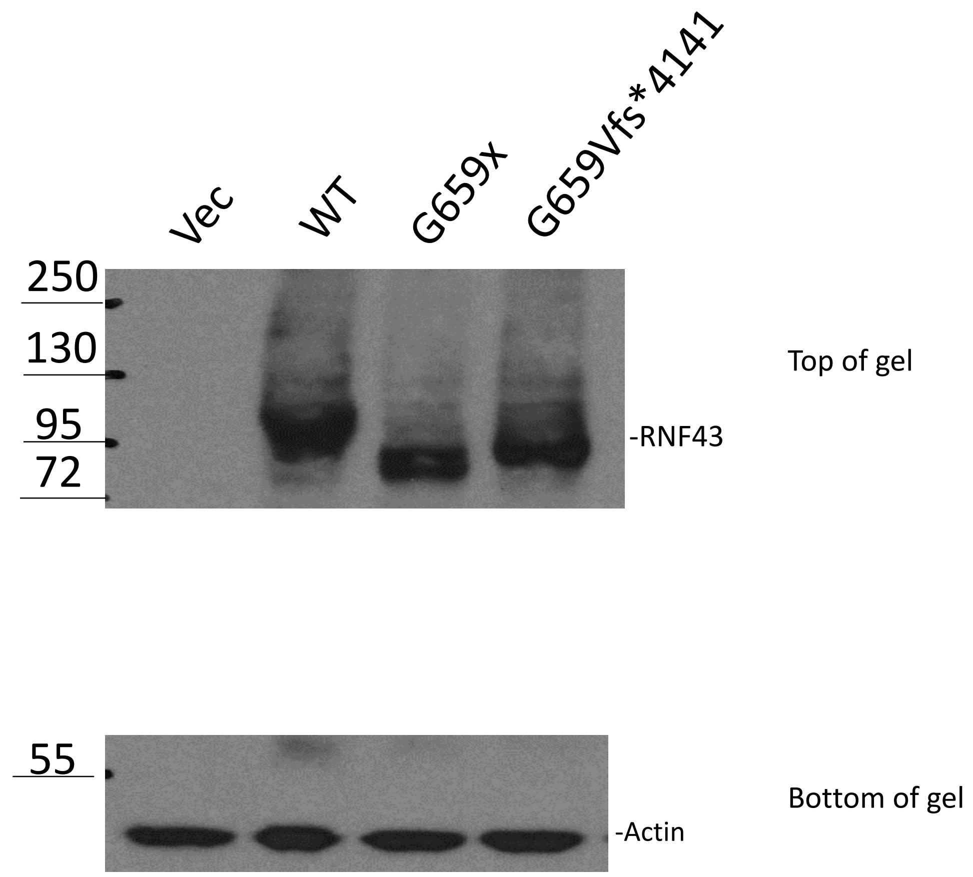

Vec
WT
G659x
G659Vfs*4141
250
130
95
72
-RNF43
Top of gel
55
-Actin
Bottom of gel

Figure 2b

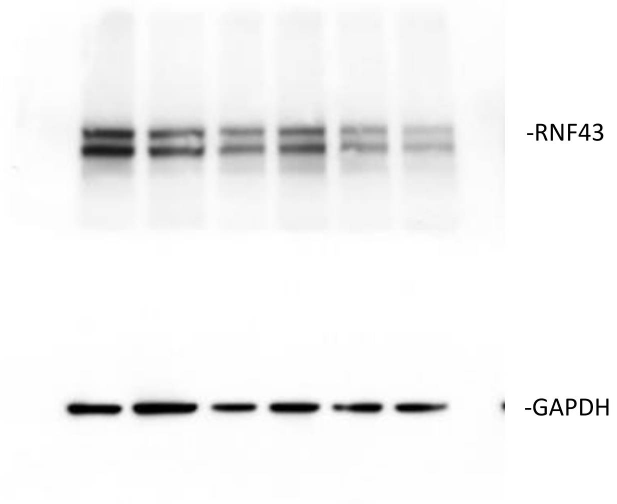

Figure 2d

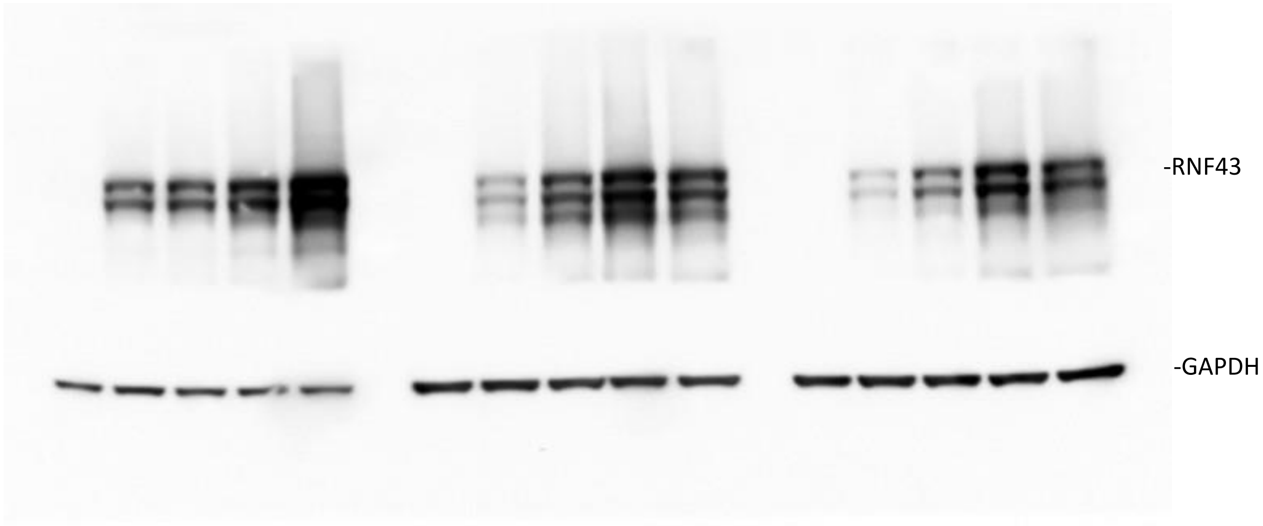

Figure 2f

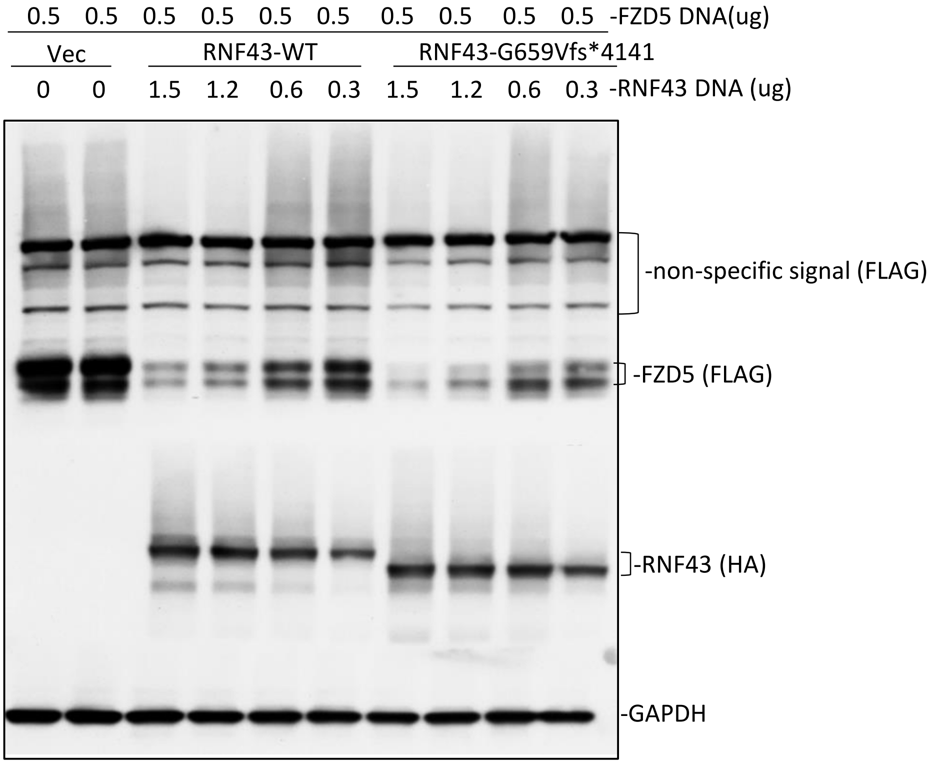
0.5 0.5 0.5 0.5 0.5 0.5 0.5 0.5 0.5 0.5 -FZD5 DNA(ug)
Vec
RNF43-WT
RNF43-G659Vfs*4141
0 0 1.5 1.2 0.6 0.3 1.5 1.2 0.6 0.3 -RNF43 DNA (ug)
-non-specific signal (FLAG)
-FZD5 (FLAG)
-RNF43 (HA)
-GAPDH

Figure 3c

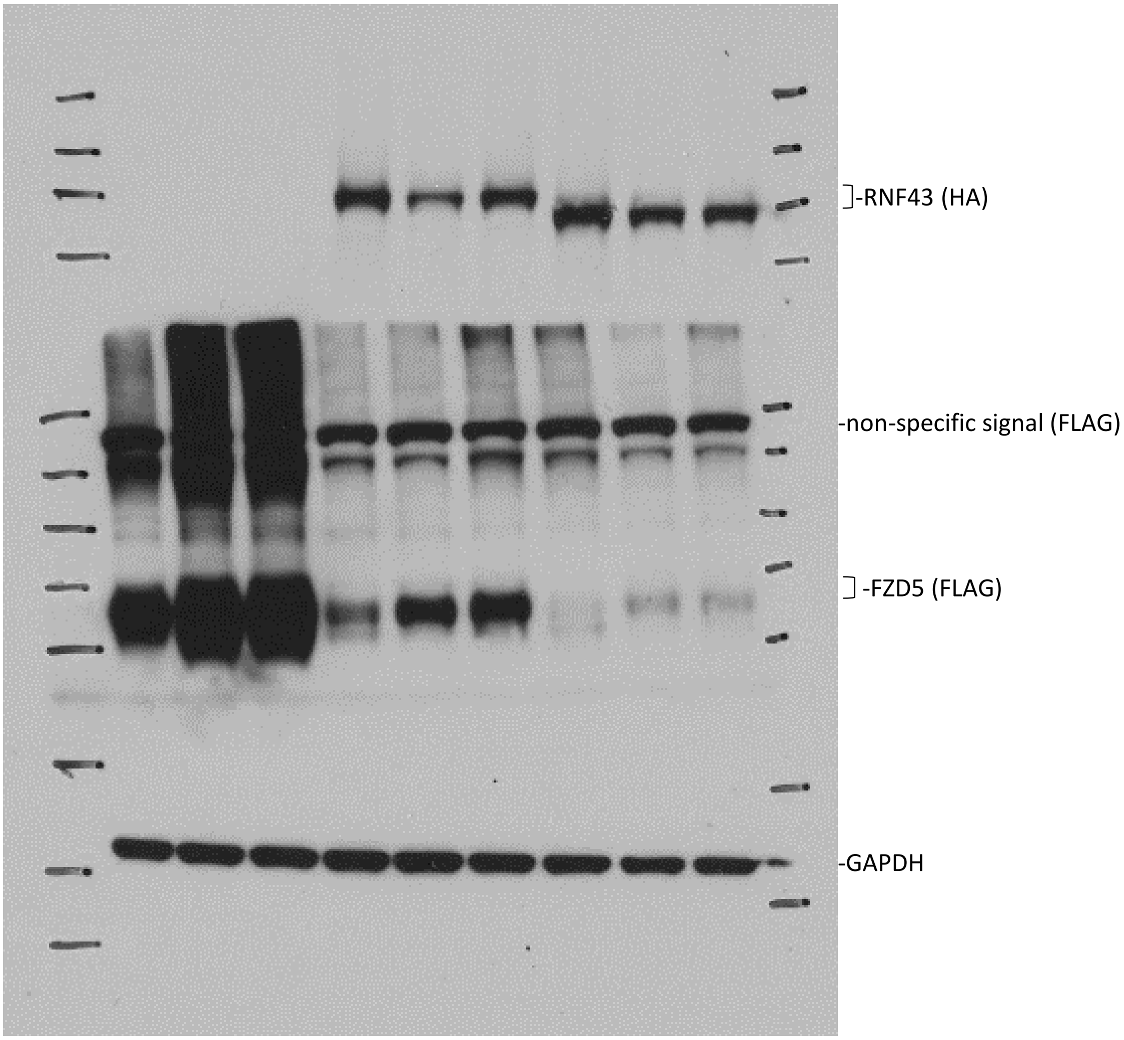

Figure 3d

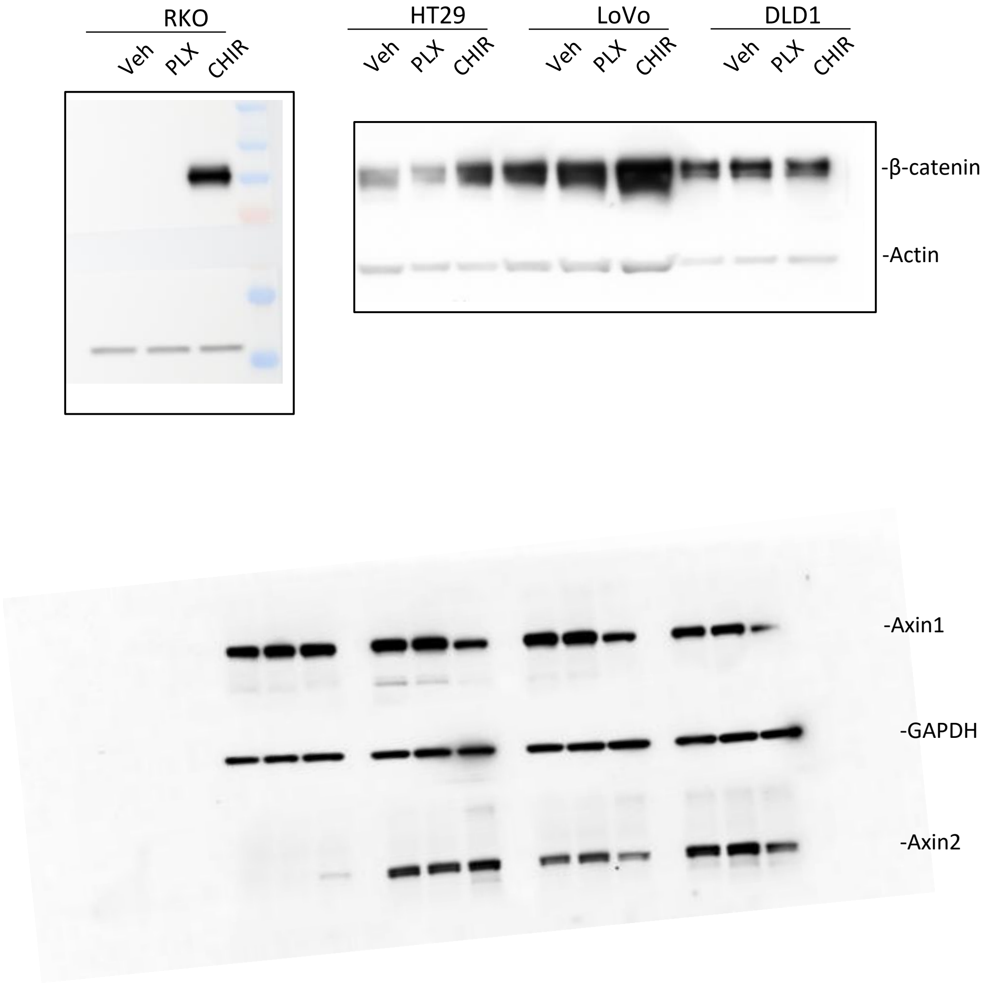
RKO
Veh
PLX
CHIR
HT29
Veh
PLX
CHIR
LoVo
Veh
PLX
CHIR
DLD1
Veh
PLX
CHIR
-β-catenin
-Actin
-Axin1
-GAPDH
-Axin2

Figure 6a

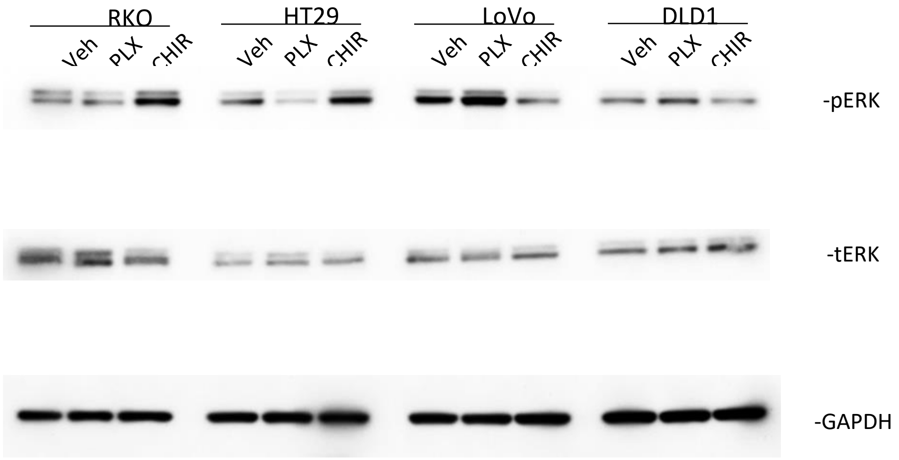
RKO
HT29
LoVo
DLD1
Veh
PLX
CHIR
Veh
PLX
CHIR
Veh
PLX
CHIR
Veh
PLX
CHIR
-pERK
-tERK
-GAPDH

Figure 6b

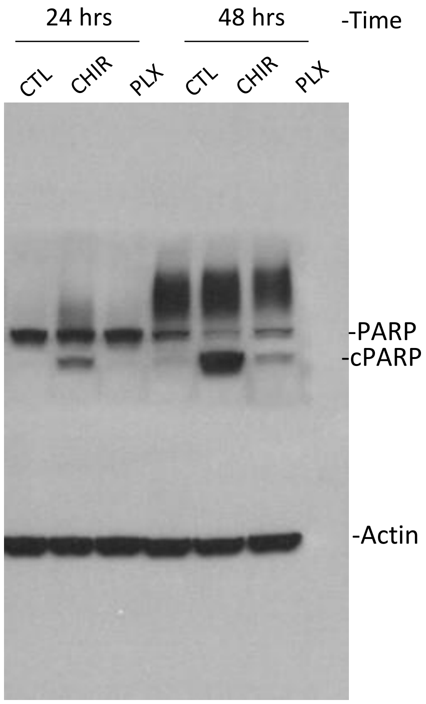

Figure 6c
